# MD Investigation on the Interaction between Carbamazepine and Two CYP Isoforms, CYP3A4 and CYP3A5

**DOI:** 10.3390/ijms24032188

**Published:** 2023-01-22

**Authors:** Shuhui Liu, Yang Xu

**Affiliations:** 1 Institute of Theoretical Chemistry, College of Chemistry, Jilin University, Changchun 130023, China; 2 School and Hospital of Stomatology, Jilin University, Changchun 130023, China

**Keywords:** carbamazepine, CYP3A4, CYP3A5, molecular dynamic simulation, interaction

## Abstract

Carbamazepine (CBZ), a commonly prescribed antiepileptic drug, in human liver, is mainly metabolized by two isoforms of cytochrome P450 (CYP), CYP3A4 and CYP3A5. Therefore, the binding of CBZ with these two enzymes plays crucial role in the biotransformation of the drug into its active metabolite. In the present work, classical molecular dynamics (MD) simulation was used to investigate the detailed interaction mechanism between CBZ and these two CYP isoforms at the atomic level. The results revealed that although CBZ can bind with the two proteins, all kinds of the interactions, including hydrogen bonds, salt bridges, hydrophobic interaction, and π-π interaction, are isoform specific. The specificity directly leads to a binding environment difference at the active sites of the two isoforms, as represented by the electrostatic surface potential maps, which further results in the varied dynamic behavior of CBZ in the two isoforms. Our research will help to deepen the understanding of the physiological functions of CYP isoforms and opens the door for the rational design and development of isoform-specific inhibitors.

## 1. Introduction

As a well-known prescribed anticonvulsant medication, carbamazepine (CBZ) has been widely used for the treatment of epilepsy (partial seizures and generalized tonic-clonic seizures) and trigeminal neuropathic pain in the clinic since it was marketed for the first time in 1962. Furthermore, CBZ is also an adjunctive treatment drugs for schizophrenia and acute mania, as well as a second-line drug for bipolar disorder and borderline personality disorder [1]. It has been reported that CBZ is also useful in the augmentation of antidepressant drug responses in treatment-resistant depression [2]. However, the administration of CBZ is observed to be accompanied by a series of idiosyncratic adverse reactions, including hematological disorders, blood disorders, hepatotoxicity, skin rash, and so on [3,4,5,6,7]. which are regarded as idiosyncratic hypersensitivity. As the antibodies of cytochrome P450 (CYP) are often detected in the sera of CBZ-hypersensitive patients, it is now believed that these immune symptoms associated with the clinical use of CBZ are related to the metabolism of the drug by this class of proteins [8,9,10]. Specifically, it is the reactive metabolites from the biotransformation of CBZ and their subsequent covalent adducts to human CYP that are the underlying reason for the immune response after the administration of CBZ [11].

**Figure 1:**
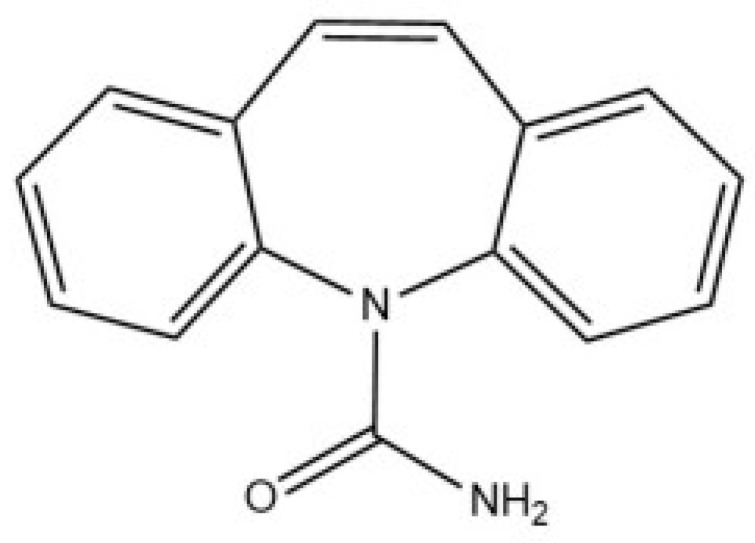
The chemical structure of CBZ 10,11-epoxide (CBZ-E).

In the human body, the main organ for CBZ metabolism is the liver which is responsible for more than 95% of the drug’s degradation [12]. Two members of the CYP3A enzyme family, CYP3A4 and CYP3A5, are the most prominent metabolic enzymes in the liver, both of which can catalyze CBZ to produce CBZ 10,11-epoxide (CBZ-E), a pharmacologically active metabolite with a potent anticonvulsant effect, through epoxidation (Figure 1) [10,13,14]. Following that, under the action of microsomal epoxide hydrolase, the drug metabolic intermediate is hydrolyzed to produce an inactive end product carbamazepine-10,11-trans dihydrodiol (CBZD), which is excreted with urine [15]. CYP3A5 is illustrated to represent more than 50% of all members in the CYP3A subfamily, while CYP3A4, the most important of the xenobiotic-metabolizing enzymes, is involved in the metabolism of more than 40% of marketed drugs and pharmaceuticals in the liver and intestine, which is more powerful than any other human P450 [16]. Furthermore, the expression levels of these two CYP isoforms are significantly higher in many tumor tissues than those in corresponding normal tissues. This kind of phenomenon in tumors means that both isoforms might have a close relation with the development, proliferation, and metastasis of tumors. For instance, the active epoxygenase activity of CYP3A4 and CYP3A5 might convert arachidonic acid to epoxyeicosatrienoic acids, further promoting the growth of breast cancer cell in a Stat3-dependent manner [17]. Some researches suggested that CYP3A5 facilitates the accumulation of the androgen receptor in the nuclear, which drives the progression of prostate cancer [18,19]. In addition, CYP3A5 is thought to impedes hepatocellular carcinomas metastasis by inhibiting the ROS/mTORC/AKT signaling pathway, which regulates the growth, survival, and migration of tumor cells [20]. From all aspects, CYP3A4 and CYP3A5 have become key targets for the design of anticancer drugs.

CYP3A4 and CYP3A5 are closely related in both structure and function [21]. First, the full-length primary sequences of two CYP isoforms have 93.8% sequene similarity. At the same time, CYP3A4 and CYP3A5 share high similarity in protein’s tertiary structures, with the RMSD (root mean square deviation) values of main-chain atoms no more than 1.4 Å, thus leading to many overlapping physiological roles. In particular, the substrate specificities for CYP3A4 and CYP3A5 isoforms significantly overlap. Moreover, CBZ is one of their common substrates. However, details of the interaction at the atomic level between CBZ and these isoforms is poorly understood. Moreover, little is known about the differences of the two isoforms in their biological function, especially the specificity of substrate recognition. In this research, conventional all-atom molecular dynamics (MD) simulation was used to unveil the differences between CYP3A4 and CYP3A5 at the atomic level in binding the same substrate, CBZ. The MD simulation revealed all the details of the interaction between the two isoforms and CBZ, from various aspects, including the hydrogen bonds, salt bridges, hydrophobic interaction, π-π interaction, and electrostatic potential surface. This present work will contribute to the rational design and development of specific inhibitors targeting individual isomerases.

## 2. Computational Details

### 2.1. Construction of the Simulation Systems

The atomic coordinates of two isoforms CYP3A4 and CYP3A5 for the construction of the simulation systems were obtained from the Protein Data Bank (www.rcsb.org (accessed on 15 May 2022)), with the corresponding PDB IDs of 5TE8 and 5VEU, respectively. The original ligands in the two crystal structures, except the heme groups, were deleted to construct the ligand-free systems of the isoform proteins. The substrate CBZ needed in the research was manully sketched in Discovery Studio 3.1 package and then docked into the active site of two isoform proteins using the CDOCKER docking algorithm, thus leading to the holo forms of two CYP proteins [22,23]. The reliability of the docking method used here was validated by re-docking the ligands in the crystal structures to the corresponding isoform proteins, which fully proves that CDOCKER algorithm is a suitable docking approach. In the docking, the substrate CBZ was kept flexible while the receptor proteins were held rigid, and all the parameters were set as the default values in the docking program, including a grid extension size of 8.0 Å, 10 random conformations with the pose cluster radius of 10 Å, and the CDOCKER scoring function based on the CHARMm force field. Prior knowledge of the binding site of the substrate in the proteins had been acquired according to the information from the experimental crystal structures, so a binding site sphere with a radius of 8 Å could be easily specified around the active site of each isoform. After that, the truncated octahedron TIP3P water box, extending up to 10 Å from the solute in each direction, was used for solvating each system [24]. The chloride ions were added into the solvent to neutralize the net charges of the solute in each system.

### 2.2. Molecular Dynamics Simulations

All the MD simulations were carried out with the Amber16 program [25]. The Amber FF14SB force field was assigned for two CYP isoform proteins [26]. The force field parameters for the substrate CBZ were generated from the generalized amber force field (GAFF), following the RESP charge calculation in the antechamber module [27]. The force field parameters of the heme group came from the existing parameter sets in the literature which was previouly optimized by Shahrokh et al. [28]. In the MD simulations, periodic boundary conditions were applied to all the systems. The cut-off distance of the short-range interaction, including the local electrostatic and van der Waals interactions, was set to 12 Å. The particle mesh Ewald (PME) method, with a grid spacing size of 1.0 Å, was used for the computation of the long-range electrostatic forces [29]. An integration time step of 2 fs was adopted and all bonds involving hydrogen atoms were constrained using the SHAKE algorithm. First, all systems were energy minimized with 20,000 steps of steepest descents, followed by 10,000 steps of conjugate gradients, by keeping the solute of each system restrained with a constraint potential of 5.0 kcal mol^−1^ Å^−2^. Next, the systems were heated to 310 K with the Langevin dynamics method and further equilibrated for 2 ns under a reference pressure of 1 bar with a modified Nosé-Hoover Langevin piston method [30,31]. After that, the constraint was gradually removed and each system was subjected to a 200 ns long MD to adequately sample the conformational space.

### 2.3. Analysis of Hydrogen Bonds

The analysis of the trajectories was performed using the CPPTRAJ program in the AMBERTOOLS package [32]. In the analysis of hydrogen bonds, the cut-off distance for the donor atom and the acceptor atom was less than 3.5 Å, and the angle formed by the donor, hydrogen, and acceptor was larger than 120°.

## 3. Results and Discussion

### 3.1. Stability of Simulation Systems

**Figure 2:**
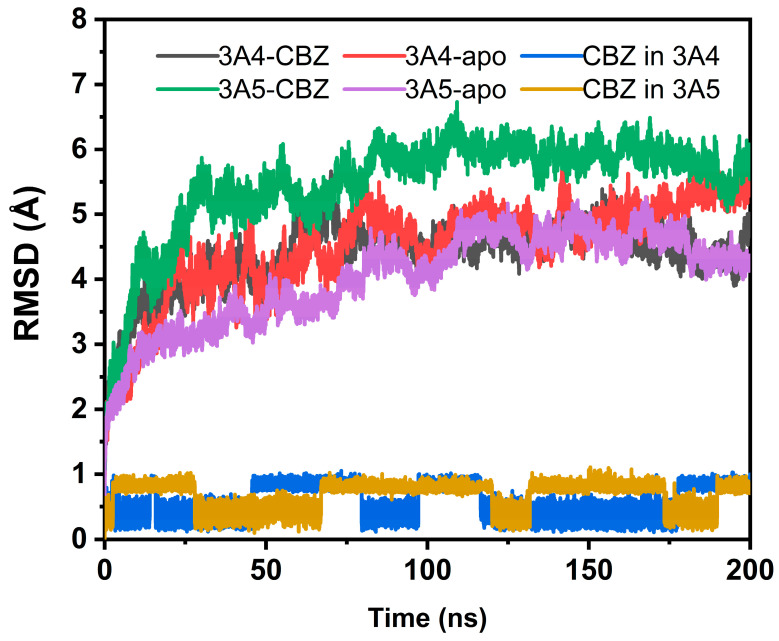
Time dependence of RMSD values in the four systems, along with the substrate CBZ.

During the simulations, the root-mean-square deviation (RMSD) values of the protein’s alpha carbon (Cα) atoms in four systems were used for the evaluation of the stability of the protein and substrate structures by taking the initial coordinates of the selected atoms in each systems as the references. The obtained RMSD profiles are shown in Figure 2. It can be seen from the figure that the overall structures of the systems, including both the protein and the substrate, were basically stable in the simulations. The stable RMSD values of three out of the four systems for the protein structure fluctuated below 5 Å. Only the protein in the CBZ and CYP3A5 complex systems displayed a larger RMSD average value, indicating the larger conformational change with respect to the initial structure. Inspection of the trajectory shows that the fluctuation of some loop regions of the protein in the MD simulation contributed to the larger RMSD values. In spite of the larger RMSD values relative to the initial coordinates, the RMSD curve of the CYP3A5-CBZ complex system gently fluctuated at the production phase of the MD simulation, indicating that the complex structure was stable. In a word, the protein structures in the four systems were in a stable state in the conformational space. For the substrate CBZ, it can be seen in Figure 2 that the RMSD values in two protein-ligand complex systems are both no more than 1 Å, illustrating that the structures of the ligand CBZ are stable in either of the complexes. However, the RMSD curves of CBZ in two complex systems show some obvious fluctuations, implying the existence of the multiple conformations of the ligand in the complex, which will be analyzed in detail later at the atomic level.

### 3.2. Flexibility of the Protein Residues in the Four Systems

**Figure 3:**
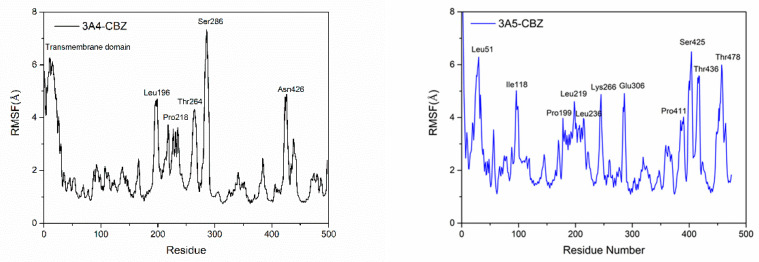
RMSF curve of all protein residues in the two complex systems, CYP3A4-CBZ (**left**) and CYP3A5-CBZ (**right**).

The effect of the substrate CBZ’s binding with the proteins on the flexibility of each protein residue could be characterized by the root-mean-square fluctuation (RMSF) of the residues (Figure 3). Comparing the RMSF values of the protein residues in two systems, it can be seen that the corresponding residues of the two proteins had different flexibility in the case of substrate binding. First, in the CYP3A4-CBZ system, the residues of the transmembrane domain had high flexibility, which can be attributed to the unfavorable effect of the solution environment in the MD simulations on the stability of the hydrophobic transmembrane residues. For the CYP3A5-CBZ complex, the residues of the corresponding part also had the significant flexibility. A different region was located near the residue Ile118, adjacent to the B’-helix of the proteins, which displayed rigidity in the CYP3A4-CBZ system but were flexible in the CYP3A5-CBZ system. As the residue Ile118 had a weak hydrophobic interaction with the substrate CBZ in CYP3A5-CBZ, it was inferred that the residue region might play an important role in the binding of CBZ to CYP3A5. For the CYP3A4 isoform, however, this region had little effect. In the CYP3A4-CBZ system, the residue Pro218 forms strong interaction with the substrate via the hydrophobic effect. However, in CYP3A5-CBZ, the residue in the corresponding site lacked a the hydrophobic interaction. They showed the same great flexibility, which is considered the common structural feature of these two CYP isoforms. In the same way, the structural flexibility of the L-helix, especially the residues 220-230, is an the inherent structural characteristic of the two proteins, because the substrate has no direct effect on the region. The residue Glu306 in CYP3A5 forms a salt bridge interaction with the substrate CBZ, so its flexibility is large in case of CBZ binding. Furthermore, the residues near the C terminal of CYP3A5 show significant fluctuation during the MD simulation, which was also different from the corresponding region of CYP3A4.

### 3.3. Dynamic Binding of CBZ to CYP3A4 and CYP3A5

**Figure 4:**
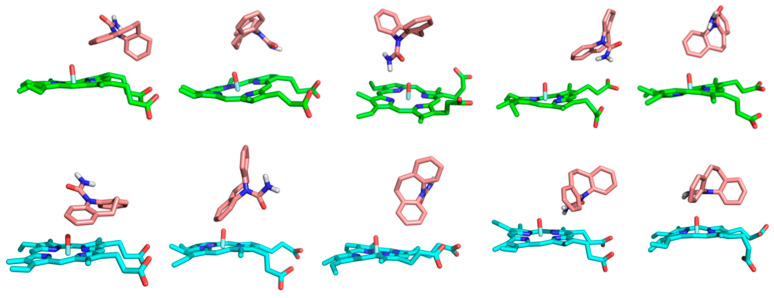
Representative conformations of the substrate CBZ in the active site of CYP3A4 (**upper**) and CYP3A5 (**lower**) during the MD simulation.

In the production phase of the conventional MD simulation, the substrate CBZ was always located in the active sites of the CYP isoforms CYP3A4 and CYP3A5 (Figure 4). It can be seen in the figure that several different representative conformations from the clustering analysis appeared in the dynamic binding of the complexes although CBZ could keep the binding in the active sites of the two isoforms. Comparing the two complexes, the conformations of the substrate CBZ in the binding site are quite similar. That is, the aromatic rings always point to the side of the protein while the hydrophilic amide part is oriented towards the heme group. The hydrophobic aromatic part of the substrate shows multiple altered conformations, which is speculated to be affected by the external solvent environment. The amide part of CBZ basically keeps its interaction with the heme group. This is very reasonable from the perspective of the physiological role of the isoforms because the heme group participates in the catalysis of the substrate. The close distance between CBZ and heme is very beneficial to the occurrence of the catalytic reaction. In addition, the similar performance of CBZ in the two isoforms reveals the general character of the substrate catalysis of CYP3A4 and CYP3A5.

### 3.4. Hydrogen Bonds and Salt Bridges in the Complex Systems

From Table 1, it can be clearly seen that the substrate CBZ formed more hydrogen bonds with CYP3A4 than CYP3A5. In the CYP3A5-CBZ complex system, only one hydrogen bond, with an occupancy of 46.38%, existed between the O17 atom of the substrate and the side chain of the CYP3A5 residue Arg106. In contrast, the CBZ molecule in the CYP3A4-CBZ complex formed three hydrogen bonds with two residues of CYP3A4, Phe108 and Arg105. Among them, the occupancy rate of the hydrogen bond between the ligand CBZ and the residue Phe108 reached 71.37%. The total occupancy between the substrate and Arg105 was 62.59%. Obviously, CBZ was more closely bound to CYP3A4 than CYP3A5, from the perspective of the hydrogen bonding. For the cofactor heme, it also hydrogen bonds with the residues Arg105 in the two isoforms. However, the total occupancy of the hydrogen bonds in the CYP3A4-CBZ complex, 143.39% (54.83% + 52.01% + 36.51%), was significantly higher than that in the CYP3A5-CBZ system, 94.01% (47.88% + 46.13%). The hydrogen bonds with the residue Trp126 had a higher proportion in CYP3A4-CBZ than in CYP3A5-CBZ. The residue Arg130 behaved similarly to the residue Arg105 in the two isoforms. Furthermore, the heme group in the CYP3A4-CBZ system formed two hydrogen bonds with the residue Arg440, while in CYP3A5 it had a hydrogen bond with the residue Arg375. In a word, the heme group has more hydrogen bond interaction in the CYP3A4-CBZ system than in CYP3A5-CBZ, suggesting that heme binds to CYP3A4 more tightly. The calculation of the binding energy between the heme and proteins also proved the difference of the affinity of the heme with the two isoforms. The average binding energy between CYP3A4 and heme was −105.8 kcal/mol while the average binding energy between heme and CYP3A5 was −69.4 kcal/mol. The protein-heme affinity in the CYP3A4-CBZ system was 36.4 kcal/mol stronger than that in the CYP3A5-CBZ complex.

In terms of the salt bridge, the substrate CBZ had no salt bridge interaction with any CYP isoform or the heme group. However, the salt bridges between some residues of the two proteins can affect the binding of the substrate CBZ in the active site. It can be seen in Table 2 that the residues Arg105 and Arg440 mentioned above formed a salt bridge with the same residue Asp123 in the CYP3A4-CBZ complex system. Here, the salt bridge network between Asp123 and Arg105 could further stabilize the hydrogen bond interaction between the substrate CBZ and the residue Arg105 and enhance the binding affinity of CBZ to the isoform CYP3A4. Futhermore, the salt bridge also helped to anchor the heme group in the binding site, which is favored to the hydrophobic and π-π interaction between the heme and the substrate CBZ. However, in the CYP3A5-CBZ complex system, the residue Asp123 had a salt bridge with the residue Arg130. The salt bridge interaction associated with Asp123 is isoform specific. Except for the two salt bridges mentioned above, there was only one salt bridge in the CYP3A4-CBZ complex which formed between the residues Asp61 and Arg372. By contrast, more salt bridge interactions existed between the related residue pairs of the protein in the CYP3A5-CBZ complex (Table 2). Some salt bridges, including those between the residue pairs Glu306-Arg105, Glu374-Arg105, Glu306-Arg106, and Glu374-Arg106, stabilized the hydrogen bonds between the substrate CBZ and the residue Arg106, increasing the binding affinity of CBZ to the isoform CYP3A5.

### 3.5. Hydrophobic and π-π Interactions Involving the Substrate in the Two Complexes

**Figure 5:**
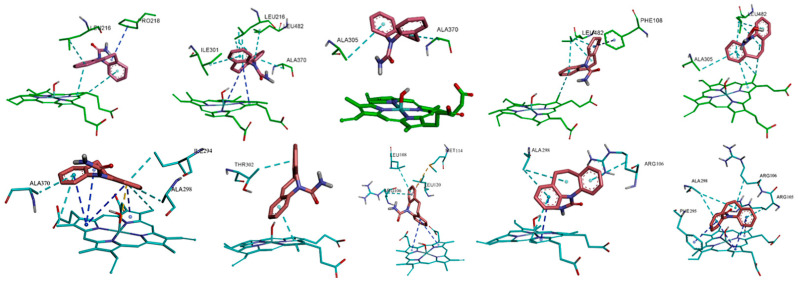
The hydrophobic interaction (dotted line colored in cyan) and π-π interaction (dotted line colored in dark blue) between the substrate CBZ and the surrounding residue in the CYP3A4-CBZ (**upper**) and CYP3A5-CBZ (**lower**) complex systems.

As the substrate CBZ molecule contains two aromatic rings, the hydrophobic interaction of the two atom groups with the surrounding environment (including protein’s residues and heme group), especially the π-π interaction, plays crucial roles in the binding of CBZ with proteins and its catalysis by the isoforms. The hydrophobic interaction, along with the π-π interactions, for the representative conformations in the dynamic binding of CBZ with the two CYP isoforms is shown in detail in Figure 5. From a comparison of the interaction of two isoforms, it can be seen that in the CYP3A4-CBZ complex system, the hydrophobic interaction between the substrate CBZ and the protein’s residues or the heme group dominated in the total interaction while the π-π interaction was much less, only existing between the substrate and the residue Phe108, the porphyrin ring of heme. This is closely related to the residue composition of the active site of CYP3A4, which is mainly composed of hydrophobic residues and therefore can not form the π-π interaction with the substrate, but has a hydrophobic interaction. The heme cofactor is likely to form the π-π interaction with the substrate CBZ because the aromatic porphyrin ring exists in the heme group. In the isoform CYP3A5, the charged amino acid residues, such as Arg105 and Arg106, appear at the active site, so the hydrophobic interaction between the substrate and the isoform is correspondingly interfered with. Hence there was not much hydrophobic interaction in CYP3A5, unlike in CYP3A4. From another point of view, due to the presence of the charged residues, the interaction between the substrate and the porphyrin ring is strengthened. Therefore, in the CYP3A5-CBZ complex system, more π-π interactions were formed between the substrate CBZ and the porphyrin ring of the heme group. From the above analysis and comparison, it can be seen that the aforementioned difference of the hydrophobic and π-π interactions between the two isoforms can be attributed to the different microenvironments of their active sites, especially the influence of the charged residues, which is consistent with the previous analysis of the hydrogen bonds and the salt bridges.

### 3.6. Analysis of Electrostatic Surface Potential

**Figure 6:**
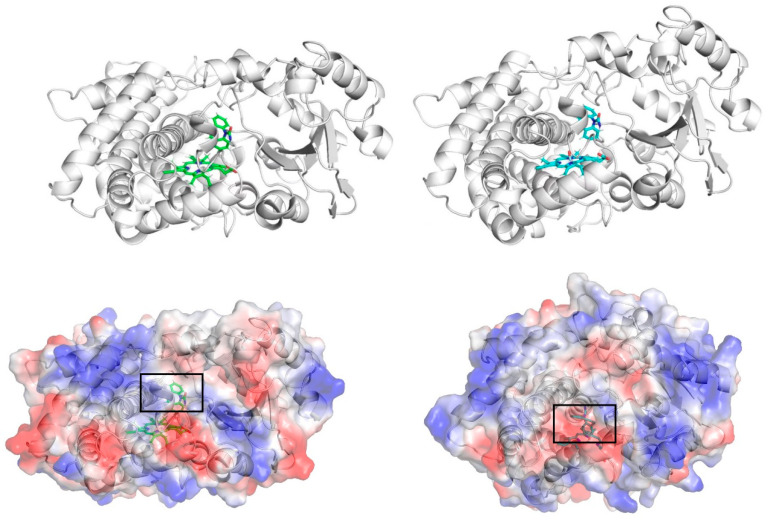
The electrostatic surface potential maps of the CYP proteins in two complex systems, CYP3A4-CBZ (**left**) and CYP3A5-CBZ (**right**).The ligand CBZ in each isoform is marked with a black square box.

From the above analysis, it can be seen that the active sites of the two isoforms have different residue composition, which gave rise to the varied behaviors of the substrate CBZ in the respective binding sites during the MD simulation. In order to further reveal these differences, the electrostatic surface potential of the CYP proteins in the two isoform-CBZ complex systems is displayed in Figure 6. According to the electrostatic surface figure, it can be seen that the substrate CBZ had different electrostatic environments when binding with the two CYP isoforms. That is, the substrate located in the positively charged environment in the CYP3A4-CBZ complex system, while in the CYP3A5-CBZ complex, the same molecule was in a negatively charged environment. These two kinds of electrostatic environment are a direct reflection of the different compositions of the active site residues, as we have previously demonstrated. In the CYP3A4-CBZ complex, the ligand CBZ concurrently forms H-bond interaction with the CYP3A4 residues Arg105 and Phe108, the proportion of which is much higher than that of the H-bond between CBZ and Arg106 in the CYP3A5-CBZ system. Furthermore, in the CYP3A4-CBZ complex, the residue Arg105, along with the residue Arg440, is anchored by the residue Asp123 via the salt bridge interaction. The existence of the two positively charged residues provides a positive environment for the binding of the substrate CBZ. By comparison, in the CYP3A5-CBZ complex system, more negatively charged residues, including Asp123, Glu306, and Glu374, form salt bridge interaction with the two positively charged residues Arg105 and Arg106, which neutralizes the positive charge in two residues, making the binding environment of CBZ more negative in the CYP3A5-CBZ complex. In this case, it is obvious that the substrate at the active sites of the two isoforms has a different dynamic behaviors.

## 4. Conclusions

Conventional molecular dynamics method was used to the research the binding of the anticonvulsant CBZ with two CYP isoforms, CYP3A4 and CYP3A5. The simulation results showed that the substrate CBZ can stably locate in the active sites of the two proteins in the whole MD stage. However, according to the atomic-level analysis, the detailed interaction between CBZ and the protein residues is different in the two isoforms, including a hydrogen bond, the salt bridge, hydrophobic interaction, and π-π interaction analysis, which also leads to the different dynamic behaviors of the substrate CBZ in two isoforms. The electrostatic surface potential maps of the proteins in two complex systems directly and significantly represent the different binding environments of the substrate CBZ in the active sites of the two isoforms. Therefore, the residue compositions in the active sites of two CYP enzymes, forming the different binding environments, is the fundamental reason for the difference of the dynamic behaviors of the substrate CBZ.

## Figures and Tables

**Table 1:** Occupancy of the hydrogen bonds in two complex systems.

3A4-CBZ	%	3A5-CBZ	%
499CBZ@O17-108PHE@H-N	71.37	476CBZ@O17-106ARG@HH11-NH1	46.38
499CBZ@O17-105ARG@HE-NE 499CBZ@O17-105ARG@HH22-NH2	32.21 30.38		
498HEM@O2A-105ARG@HH22-NH2 498HEM@O1A-105ARG@HH22-NH2 498HEM@O2D-105ARG@HH21-NH2	54.83 52.05 36.51	475HEM@O1A-105ARG@H-N 475HEM@O1A-105ARG@H-N	47.88 46.13
498HEM@O1D-126TRP@HE1-NE1 498HEM@O2D-126TRP@HE1-NE1	89.12 72.36	475HEM@O1D-126TRP@HE1-NE1 475HEM@O2D-126TRP@HE1-NE1	82.79 75.31
498HEM@O1D-130ARG@HH22-NH2 498HEM@O2D-130ARG@HH22-NH2 498HEM@O2D-130ARG@HE-NE 498HEM@O1D-130ARG@HE-NE	47.95 45.50 33.74 25.19	475HEM@O2D-130ARG@HE-NE 475HEM@O2D-130ARG@HH21-NH2 475HEM@O1D-130ARG@HH21-NH2	35.91 28.43 26.18
498HEM@O2A-440ARG@HH22-NH2 498HEM@O1A-440ARG@H22-NH2	45.28 43.29	475HEM@O2A-375ARG@HH21-NH2	28.68

**Table 2:** Salt bridge analysis in the complex systems.

3A4-CBZ	%	3A5-CBZ	%
123ASP@OD2-105ARG@HH12-NH1 123ASP@OD2-105ARG@HH11-NH1 123ASP@OD1-105ARG@HH12-NH1 123ASP@OD1-105ARG@HH11-NH1 123ASP@OD2-105ARG@HH22-NH2 123ASP@OD2-105ARG@HH21-NH2	32.88 32.88 27.31 27.31 21.75 21.75	306GLU@OE2-105ARG@HH22-NH2 306GLU@OE2-105ARG@HH21-NH2 306GLU@OE1-105ARG@HH22-NH2 306GLU@OE1-105ARG@HH21-NH2 306GLU@OE1-105ARG@HH12-NH1 306GLU@OE1-105ARG@HH11-NH1 306GLU@OE2-105ARG@HH12-NH1 306GLU@OE2-105ARG@HH11-NH1	99.62 99.62 99.31 99.31 98.62 98.62 98.50 98.50
61ASP@OD2-372ARG@HH22-NH2 61ASP@OD2-372ARG@HH21-NH2 61ASP@OD2-372ARG@HE-NE 61ASP@OD1-372ARG@HH22-NH2 61ASP@OD1-372ARG@HH21-NH2 61ASP@OD1-372ARG@HE-NE	100.00 100.00 100.00 100.00 100.00 99.81	374GLU@OE1-105ARG@HH22-NH2 374GLU@OE1-105ARG@HH21-NH2 374GLU@OE2-105ARG@HH22-NH2 374GLU@OE2-105ARG@HH21-NH2 374GLU@OE1-105ARG@HE-NE 374GLU@OE2-105ARG@HE-NE	99.19 99.19 97.69 97.69 93.81 91.37
123ASP@OD2-440ARG@HH12-NH1 123ASP@OD2-440ARG@HH11-NH1 123ASP@OD1-440ARG@HH12-NH1 123ASP@OD1-440ARG@HH11-NH1 123ASP@OD2-440ARG@HH22-NH2 123ASP@OD2-440ARG@HH21-NH2 123ASP@OD1-440ARG@HH22-NH2 123ASP@OD1-440ARG@HH21-NH2	65.19 65.19 64.56 64.56 63.81 63.81 63.81 63.81	301GLU@OE2-106ARG@HH22-NH2 301GLU@OE2-106ARG@HH21-NH2 301GLU@OE2-106ARG@HH12-NH1 301GLU@OE2-106ARG@HH11-NH1 301GLU@OE1-106ARG@HH22-NH2 301GLU@OE1-106ARG@HH21-NH2 301GLU@OE1-106ARG@HH12-NH1 301GLU@OE1-106ARG@HH11-NH1	54.00 54.00 52.44 52.44 49.06 49.06 46.00 46.00
		306GLU@OE2-106ARG@HH22-NH2 306GLU@OE2-106ARG@HH21-NH2 306GLU@OE1-106ARG@HH22-NH2 306GLU@OE1-106ARG@HH21-NH2 306GLU@OE2-106ARG@HH12-NH1 306GLU@OE2-106ARG@HH11-NH1 306GLU@OE1-106ARG@HH12-NH1 306GLU@OE1-106ARG@HH11-NH1	66.00 66.00 60.87 60.87 42.12 42.12 38.62 38.62
		374GLU@OE2-106ARG@HE-NE 374GLU@OE1-106ARG@HE-NE 374GLU@OE2-106ARG@HH22-NH2 374GLU@OE2-106ARG@HH21-NH2 374GLU@OE1-106ARG@HH22-NH2 374GLU@OE1-106ARG@HH21-NH2	29.69 28.44 23.87 23.87 20.56 20.56
		123ASP@OD1-130ARG@HH22-NH2 123ASP@OD1-130ARG@HH21-NH2 123ASP@OD2-130ARG@HH22-NH2 123ASP@OD2-130ARG@HH21-NH2 123ASP@OD1-130ARG@HH12-NH1 123ASP@OD1-130ARG@HH11-NH1 123ASP@OD2-130ARG@HH12-NH1 123ASP@OD2-130ARG@HH11-NH1	36.56 36.56 34.31 34.31 33.19 33.19 32.25 32.25

## Data Availability

All the simulation result and data requests can be directed to the corresponding author.
